# Gender Differences in Neural Responses to Perceptually Invisible Fearful Face—An ERP Study

**DOI:** 10.3389/fnbeh.2017.00006

**Published:** 2017-01-26

**Authors:** Seung A. Lee, Chai-Youn Kim, Miseon Shim, Seung-Hwan Lee

**Affiliations:** ^1^Clinical Emotion and Cognition Research Laboratory, Inje UniversityGoyang, South Korea; ^2^Department of Psychology, Korea UniversitySeoul, South Korea; ^3^Department of Biomedical Engineering, Hanyang UniversitySeoul, South Korea; ^4^Department of Psychiatry, Inje University Ilsan Paik HospitalGoyang, South Korea

**Keywords:** gender difference, emotional processing, subthreshold, fearful face, event-related potential

## Abstract

Women tend to respond to emotional stimuli differently from men. This study aimed at investigating whether neural responses to perceptually “invisible” emotional stimuli differ between men and women by exploiting event-related potential (ERP). Forty healthy participants (21 women) were recruited for the main experiment. A control experiment was conducted by excluding nine (7 women) participants from the main experiment and replacing them with additional ten (6 women) participants (total 41 participants) where Beck's Anxiety Inventory (BAI) and Beck's Depression Inventory (BDI) scores were controlled. Using the visual backward masking paradigm, either a fearful or a neutral face stimulus was presented in varied durations (subthreshold, near-threshold, or suprathreshold) followed by a mask. Participants performed a two-alternative forced choice (2-AFC) emotion discrimination task on each face. Behavioral analysis showed that participants were unaware of masked stimuli of which duration was the shortest and, therefore, processed at subthreshold. Nevertheless, women showed significantly larger response in P100 amplitude to subthreshold fearful faces than men. This result remained consistent in the control experiment. Our findings indicate gender-differences in neural response to subthreshold emotional face, which is reflected in the early processing stage.

## Introduction

Emotional information carries biological and social significance in our daily lives from when we attempt to avoid a life-threatening danger to when we try to understand others. How we process the emotional information is closely related to how we interpret other's intentions and how we behave. Moreover, identical emotional information could affect each of us differently. Individual difference in processing emotion could explain the mechanism of emotion processing. There are several factors known to affect the emotion processing such as personality traits, experiences, or genetics (Hamann and Canli, 2004). For example, extravert personality trait showed positive correlation with the degree of activation in the amygdala, which is known to be involved in emotion processing, when happy faces were viewed (Canli et al., 2002). Activity in the amygdala was also correlated positively with pessimism while unpleasant stimuli such as snakes were viewed (Fischer et al., 2001). In another study, differences in voluntary emotional regulation across individuals evoked by emotional visual stimuli modulated activation in the amygdala (Schaefer et al., 2002). Moreover, people with a genotype such as 5-HTT showed enhanced activity in the amygdala when they view faces with emotional expressions (Hariri et al., 2002).

Gender is another source of individual differences in response to emotional information (Bradley et al., 2001; Kret and De Gelder, 2012). Several studies have reported gender differences in trait and neural responses to emotional stimuli. Women were found to be more expressive as shown by more responsive electromyography (EMG) to emotional face stimuli (Dimberg and Lundquist, 1990) and greater skin conductance response (SCR) to emotional films (Kring and Gordon, 1998). Several studies have demonstrated that women show higher sensitivity in recognizing emotional information presented not only in visual modality (Kring and Gordon, 1998; Montagne et al., 2005; Collignon et al., 2010) but also in auditory modality (Collignon et al., 2010). Women's sensitivity to emotional information is further supported by higher accuracy in emotional recognition and categorization (Thayer and Johnsen, 2000; Hall and Matsumoto, 2004). In response to emotional stimuli, women tend to show facial expressions more accurately labeling the emotional contents (Wagner et al., 1986), and different patterns of SCR (Kring and Gordon, 1998) and startle reflex (Bianchin and Angrilli, 2012) to emotional stimuli compared with men.

Some studies have reported that this sensitivity to emotional information in women may explain why women are more likely to suffer from psychiatric problems than men (McGrath et al., 1990; Nolen-Hoeksema and Girgus, 1994). There are many studies that found women to be more sensitive and responsive to unpleasant stimuli such as sad faces while men are more sensitive to stimuli with positive valence (Williams and Gordon, 2007; Li et al., 2008). Gasbarri et al. (2007) have reported that women are more selectively attentive to biologically relevant stimuli with negative emotional valence such as sadness and fear. Bradley et al. (2001) have found that women react more defensively as observed by unpleasant facial muscle activity (EMG), cardiac deceleration, and startle reflex to unpleasant stimuli, which all reflect intensified selective attention. Lang et al. (1998) observed enhanced activation in the occipital cortex in response to unpleasant stimuli in women and pleasant stimuli in men.

Studies using event-related potentials (ERPs) have reported gender differences in neural responses to perceptually visible emotional stimuli, which is reflected in enhanced activity in women compared with activity in men. The gender-related ERP differences have been observed in both early and late responses. For example, N200 component was enhanced more when women viewed unpleasant stimuli compared to when men viewed the same stimuli (Lithari et al., 2010). Campanella et al. (2004) found using the oddball paradigm that N2b latency differentiated happy and fearful stimuli in men while such differentiation was not found in women. In another study, women showed more enhanced P2, the component implicated in higher-order attentional processing, compared to men in response to unanticipated negative stimuli in a modified cue-target paradigm (Jin et al., 2013). These results all support that women have attentive bias to emotional stimuli, particularly to unpleasant stimuli.

Enhanced responses in late components such as P300 and LPP are also observed in women (Oliver-Rodríguez et al., 1999; Gasbarri et al., 2006, 2007; Han et al., 2008). Gasbarri et al. (2006) observed that women showed larger P300 responses and better memory retrieval for emotional stimuli than men. Difference in even later component, P450, was observed in Orozco and Ehlers's (1998) study in which women showed overall longer latency and higher amplitude in response to both happy and sad faces. In another study by Luo et al. (2014), only women showed longer latency of late positive potential (LPP) to moderate negative stimuli drawn from International Affective Picture System (IAPS) despite no gender difference in early responses.

Taken together, these studies suggest that women show higher sensitivity and more responsiveness when they view emotional stimuli of negative valence, which might be the source of the tendency that women are more vulnerable to affective disorders such as anxiety and depression compared to men (Nolen-Hoeksema, 1987). It is noteworthy that in most of the previous studies, emotional information was delivered to the participants with clear visibility by presenting the stimuli for long duration with full contrast. However, emotional information can also be fleeting and feeble. A considerable amount of evidence has shown that emotional information can be processed without conscious awareness, indicative of behavioral and neurophysiological responses to perceptually *invisible* emotional stimuli (Tamietto and de Gelder, 2010). However, few studies have investigated whether men and women differ in their response to emotional stimuli. Among those few studies, Hall and Matsumoto (2004) showed women's superior ability recognizing emotional facial expressions even when the stimuli were presented so fast as to be “at the edge of conscious awareness.” Specifically, they presented each face in one of six expressions (anger, contempt, disgust, fear, happiness, sadness, or surprise) briefly (70, 130, or 200 ms) in the middle of 1-s presentation of a neural face of the same person. As in the prolonged viewing condition, women were more accurate than men recognizing each briefly presented expression. More recently, Donges et al. (2012) employed a version of subliminal affective priming procedure where the valence of a subliminally processed emotional facial expression influences the valence of a subsequent target to examine gender difference in response to invisible emotional stimuli. The results showed that despite the lack of conscious awareness of the briefly presented and masked happy prime face (30 ms), women judged the subsequent neutral face more positively compared to men. Interestingly, such gender difference in subliminal affective priming was not significant in the case of sad prime face. Based on those limited number of recent studies, it is important to investigate the gender differences in neurophysiological responses to perceptually *invisible* emotional stimuli, to further support the association between women's vulnerability to affective disorder and the gender difference in emotion processing below conscious awareness.

In the current work, we set out to examine whether there exists gender differences in response to emotional stimuli processed outside conscious awareness by rendering the stimuli perceptually *invisible*. For that, we used a backward masking paradigm to render a fearful or neutral face invisible while measuring ERP responses to it. The duration of the masked face was varied, which enabled us to compare ERP responses to the subthreshold emotional stimuli with ERP responses to stimuli processed at near-threshold and suprathreshold. ERP components of interest included P100, N170, early posterior negativity (EPN), N250, and P300, which have been implicated in affective face processing in a range of stages. We also took other personality factors into consideration by including a battery of questionnaires such as State and Trait Anxiety Inventory-trait (STAI-trait; Spielberger, 1970), Positive and Negative Affect Schedule (PANAS; Watson et al., 1988), Beck's Anxiety Inventory (BAI; Beck et al., 1988a), and Beck's Depression Inventory (BDI; Beck et al., 1988b). To disentangle neuroticism factor from the gender differences, we performed an additional, control experiment by including participants whose BAI and BDI scores are within healthy ranges. This consideration was particularly important due to several reasons; First, gender difference in emotion processing may be partly related to the higher incidence of neuroticism in women than in men (Kessler et al., 2005). Second, women's relative vulnerability to neuroticism may be related to involuntary and automatic bias toward emotions (Mayer and Merckelbach, 1999). Last but not least, a recent study showed the interaction between gender and neuroticism factors in disengaging attention from the location of *invisible* fearful faces (Tan et al., 2011). In the main and the control experiments, we hypothesized that the early response to fearful faces processed at subthreshold would be enhanced for women compared to men as observed in P100, EPN, or N250 components, which are relevant to low-level perceptual processes. The later response to consciously, or fearful faces processed at suprathreshold would be enhanced for women compared to men as observed in P300 or N170 components, relevant to higher-level perceptual processes of emotional faces.

## Materials and methods

### Participants

Fourty participants (19 men) volunteered for the study through online recruitment. We based the number of participants required for the experiment on previous studies that investigated gender difference by measuring ERP response (Gasbarri et al., 2006, *n* = 48; Lithari et al., 2010, *n* = 28; Orozco and Ehlers, 1998, *n* = 35). There was no statistically significant gender difference of age [men: Mean = 29.89 years, standard deviation (*SD*) = 7.03; women: Mean = 31.57 years, *SD* = 8.11] and education (men: Mean = 13.58 years, *SD* = 1.71; women: Mean = 14.19 years, *SD* = 1.4). All participants were right-handed, as determined by asking about the hand used for scissors. All had normal or corrected-to-normal visual acuity. Those who have a history of neurological or psychiatric disorders were excluded from the study because these factors are known to effect the sensitivity to emotional information (Surguladze et al., 2004; Bar-Haim et al., 2005). Anxiety and depressive symptoms were examined by self-report scales: STAI-trait, PANAS, BAI, and BDI. The demographic characteristics of the participants are shown in Table 1. All participants provided written informed consent, which was approved by the Institutional Review Board of Inje University Ilsan Paik Hospital.

**Table 1:** **Demographic characteristics and questionnaire scores of participants in the main experiment**.

	**Men (*n* = 19)**	**Women (*n* = 21)**	***t*-Value**	***p* value**
Age (years)	29.89(±7.03)	31.57(±8.11)	−0.695	0.49
Education (years)	13.58(±1.71)	14.19(±1.4)	−1.242	0.22
STAI-trait	40.89(±5.17)	44.62(±4.75)	−2.372	0.023^*^
PANAS Score				
Positive	19.21(±4.61)	20.05(±5.66)	−0.153	0.422
Negative	21.16(±5.4)	22.48(±4.86)	−0.812	0.614
BAI	3.26(±4.38)	8.86(±7.17)	−3.005	0.005^**^
BDI	5.68(±5.5)	10.9(±8.06)	−2.366	0.023^*^

*Values are the mean (±standard deviation) of each gender group*.

* *p < 0.05*,

** *p < 0.01. STAI_trait, State and Trait Anxiety Inventory_trait; PANAS, Positive and Negative Affect Schedule; BAI, Beck's Anxiety Inventory; BDI, Beck's Depression Inventory*.

### Stimuli and apparatus

A total of 24 face images were chosen from the Korean Facial Expressions of Emotion (KOFEE) stimuli set (Park et al., 2011). The selected images included four women and four men with facial expressions of fearful and neutral and were used as target stimuli. Additional images including three women and three men with neutral facial expression were selected for mask stimuli. Background, hair, and facial contours were removed from the selected face images using Photoshop (Photoshop CS6). Contrast and luminance of stimuli were matched using Matlab, (2008a; The MathWorks, Natick, MA) in conjunction with Psychophysics Toolbox ver. 3.0.1 (Brainard, 1997; Pelli, 1997). For the mask stimuli, the six images were spatially scrambled so that local image features were maintained yet face recognition was not possible.

Stimuli were presented on a 17-inch CRT monitor (Samsung CD197GP; 85-Hz refresh rate). Display monitor was situated 1 m away in front of the participants and subtended a maximum visual angle of 4° × 4°.

### Procedures

**Figure 1:**
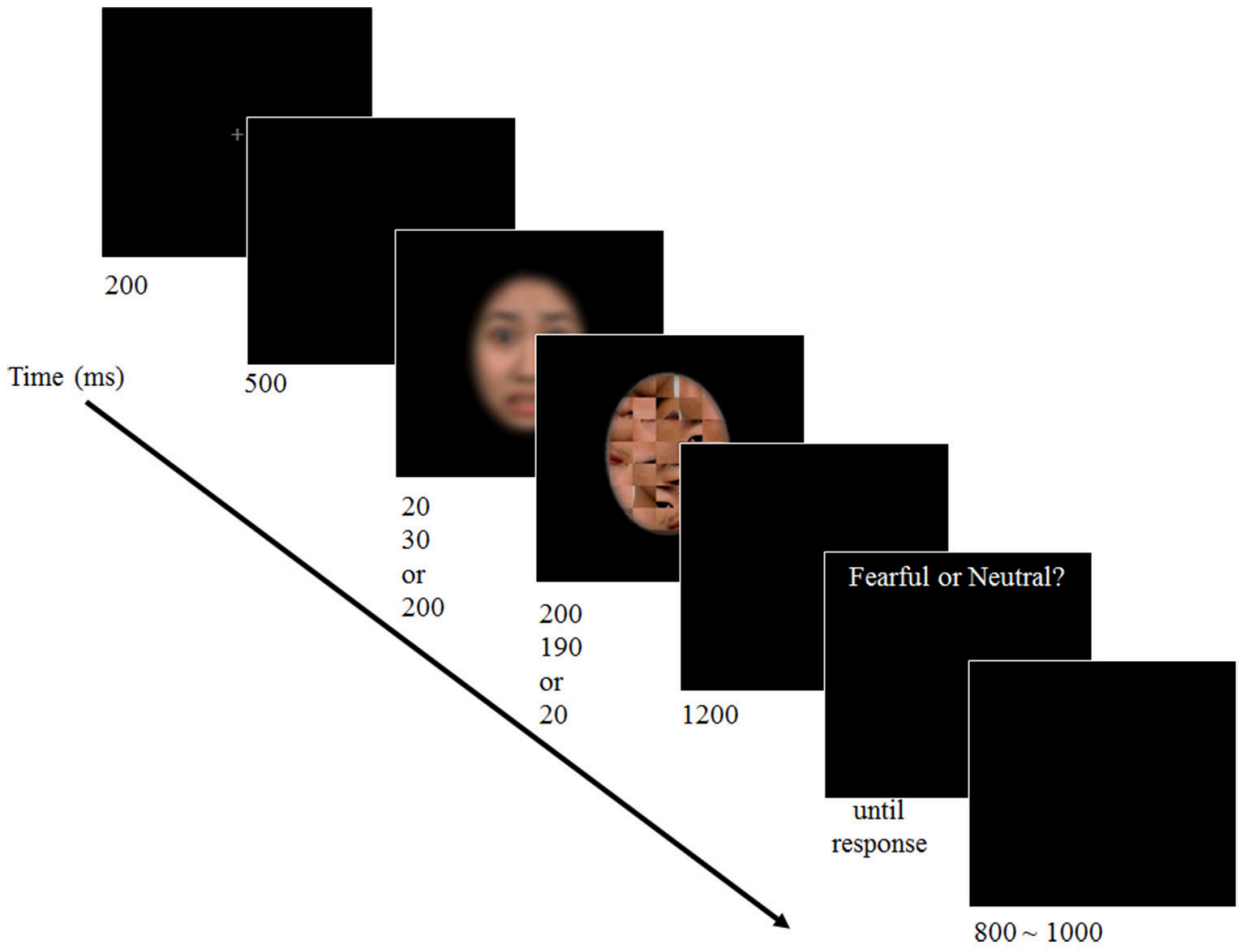
**Schematics of the experimental procedure**. Participants performed a 2-AFC emotion discrimination task upon viewing a face with varied duration presented in a backward masking paradigm. (The facial image of the stimuli is blurred here to protect the identity of the actor).

Participants went through the preparation procedure for an ERP experiment for ~15 min. When ready, they pressed the spacebar to begin the experiment. Break time was given every 5 min and participants pressed the spacebar when they were ready to continue. A trial began 30 s after the spacebar press to allow participants to be ready and to ensure stabilization of brain waves. The trial began with 200-ms presentation of a fixation cross. A blank screen was presented for 500 ms followed by a target face and a mask. The duration of a target face was manipulated in three levels, i.e., 20 ms (subthreshold), 30 ms (near-threshold), or 200 ms (suprathreshold). The duration of a mask stimulus was 200, 190, or 20 ms, respectively. Therefore, the total duration of target and mask faces was constant as 220 ms. After 1200 ms of another blank screen, participants responded whether the presented target face was fearful or neutral. Participants were allowed to take as much time as needed. After the response was made, a next trial began with random inter-trial interval (ITI) of 800–1000 ms (see Figure 1). It took ~40 min to complete the experiment.

There were a total of 384 trials; 128 trials for each of the three awareness—i.e., subthreshold, near-threshold, and suprathreshold—conditions. For each condition, the target stimulus for a half of the trials was neutral while the other half was fearful.

### EEG recording and analyses

Electroencephalography (EEG) was synchronized to the onset of stimulus presentation using Matlab. EEG activity was recorded and amplified using a Neuroscan NuAmps amplifier (Compunedics USA, El Paso, TX, ISA). Recording sites included 62 scalp positions (FP1, FPz, FP2, AF3, AF4, F7, F5, F3, F1, Fz F2, F4, F6, F8, FT7, FC5, FC3, FC1, FCz, FC2, FC4, FC6, FT8, T7, C5, C3, C1, Cz, C2, C4, C6, T8, TP7, CP5, CP3, CP1, CPz, CP2, CP4, CP6, TP8, P7, P5, P3, P1, Pz, P2, P4, P6, P8, PO7, PO5, PO3, POz, PO4, PO6, PO8, CB1, O1, Oz, O2, and CB2). The vertical electro-oculogram (EOG) was recorded with additional two electrodes, one located above, and one below the left eye. The horizontal EOG was recorded at the outer canthi of each eye. The signals were referenced to Cz and the ground electrode was at the forehead. EEG data were recorded at 1000 Hz sampling rate with a 0.1–100 Hz band-pass filter.

EEG data were initially processed using Scan 4.3. and re-referenced offline to the average reference. Eye blinks were removed from the data using established mathematical procedures (Semlitsch et al., 1986). Trials including significant physiological artifacts (amplitude exceeding ±70 uv) were rejected. Furthermore, gross artifacts such as movements were excluded by visual inspection. After the artifact removal, baseline correction was conducted by subtracting the mean of 200-ms pre-stimulus data from the mean of 900-ms post-stimulus data for each trial. Data were band-pass filtered at 1–30 Hz then epoched between 200-ms pre-stimulus and 900-ms post-stimulus.

The sufficient number of accepted ERP epochs was obtained for all conditions and the average acceptance rate did not differ significantly between conditions [subthreshold fearful: men 57.78 ± 6.33 (*SD*), women 55.76 ± 10.04; subthreshold neutral: men 57.52 ± 6.94, women 56.71 ± 9.23; near-threshold fearful: men 57.1 ± 6.27, women 56.47 ± 8.78; near-threshold neutral: men 57.36 ± 7.08, women 55.95 ± 8.9; suprathreshold fearful: men 58.15 ± 6.5, women 56.8 ± 9.35; suprathreshold neutral: men 58.05 ± 6.3, women 56.8 ± 7.82].

A grand-average waveform for each electrode within each group was obtained by averaging all epochs within each participant and then across all the participants. To determine the time windows for peak detection, we analyzed the mean global field potential (GFP) for each ERP component on grand averaged data across the conditions in all of the participants (Hamburger and vd Burgt, 1991). The final time windows were determined based on the maximal time window from scalp topography of GFP and from previous studies. The target components for the present study were determined as follows: P100 (50–150 ms at O1 and O2), EPN (150–300 ms at O1 and O2), N170 (110–210 ms at PO7 and PO8), N250 (160–360 ms at C3, Cz, and C4), and P300 (300–450 ms at FC3, FCz, and FC4).

### Statistical analyses

Independent *t*-tests were conducted to compare age, education, and scores from the anxiety and depression questionnaires between men and women. Behavioral accuracy for the discrimination task was analyzed with the gender as between-participant factor and awareness (subthreshold, near-threshold, suprathreshold) as within-participant factor. For ERP data analyses, a four-way mixed ANOVA was conducted with gender (men, women) as between-participant factor and awareness (subthreshold, near-threshold, suprathreshold), emotion (fearful, neutral), and electrode position according to the target components and hemisphere (right and left for P100, EPN, and N170, and right, center and left for N250 and P300) as within-participant factors. In behavioral and ERP statistical analysis, Bonferroni method was used in *post-hoc* testing of multiple ANOVA interactions.

## Results

### Anxiety/depression questionnaires

There were significant difference between men and women in the scores of the anxiety and depression questionnaires including STAI-trait [*t*_(38)_ = −2.372, *p* < 0.05], BAI [*t*_(38)_ = −3.005, *p* < 0.01], and BDI scores [*t*_(38)_ = −2.366, *p* < 0.05; Table 1]. Scores of PANAS (either positive or negative) did not differ between men and women [positive: *t*_(38)_ = −0.153, *p* = 0.422; negative: *t*_(38)_ = −0.812, *p* = 0.614]. STAI-trait, BAI, and BDI scores were considered as covariates in the subsequent behavioral and ERP data analyses.

### Behavioral results

**Figure 2:**
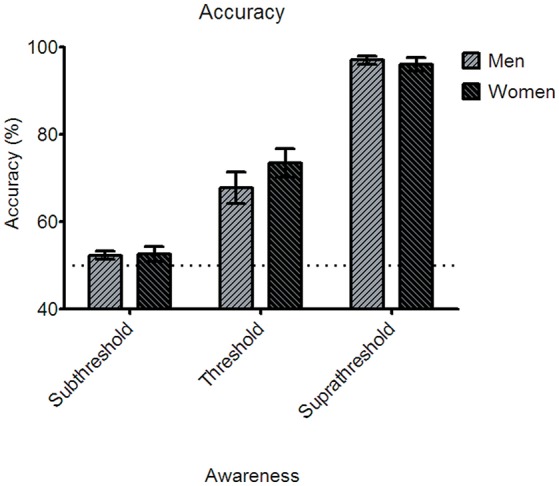
**Behavioral accuracy for the three awareness conditions in the main experiment**. None of the three awareness conditions showed statistically significant gender differences of the accuracy. Error bars denote ±1 standard error of mean. Note that the accuracy in the subthreshold condition was not different statistically from the chance level in both the gender groups.

The behavioral performance indicated by emotion discrimination accuracy for the subthreshold condition was 52.34 ± 0.94% (Standard error of mean: SEM) for men and 52.64 ± 1.65% for women. For the near-threshold condition, men performed at the accuracy of 67.8 ± 3.67% and women at 73.47 ± 3.27%. In the suprathreshold condition, men's accuracy was 97 ± 0.9%, and women's accuracy was 96 ± 1.52% (see Figure 2).

A two-way mixed ANOVA analysis was conducted to examine whether the paradigm's manipulation of awareness was successful. The analysis showed the main effect of awareness [*F*_(2, 76)_ = 243.96, *p* < 0.001], which suggests that the performances for three conditions of awareness differ from each other and the awareness of the emotional facial stimuli is successfully manipulated. Further, one-sample *t*-test was conducted to examine whether the awareness of the subthreshold condition was indeed processed subliminally and thus the performance is at the chance level. The results showed that the accuracy in the subthreshold condition did not differ significantly from the 50% chance level [men: *t*_(18)_ = 1.425, *p* = 0.171; women: *t*_(20)_ = 0.99, *p* = 0.334]. This result indicates that the participants made random responses in the emotion discrimination task due to the “invisibility” we purposefully introduced.

A two-way mixed ANOVA analysis further showed that neither the main effect of gender [*F*_(1, 38)_ = 0.565, *p* = 0.457] nor the interaction between awareness and gender [*F*_(2, 76)_ = 1.525, *p* = 0.224] was significant statistically (*Bonferroni corrected*). *Post-hoc* paired *t*-tests showed significant gender difference in none of the awareness conditions [subthreshold, *F*_(1, 39)_ = 0.023, *p* = 0.88; near-threshold, *F*_(1, 39)_ = 1.336, *p* = 0.255; suprathreshold, *F*_(1, 39)_ = 0.248, *p* = 0.621]. In other words, awareness did not differ between men and women in discriminating fearful and neutral faces in all three stimulus durations that induced subthreshold, near-threshold, and suprathreshold processing of the faces.

### ERP results

#### P100

Turning now to the ERP results, the main effect of gender was significant statistically [*F*_(1, 35)_ = 4.561, *p* < 0.05]. *Post-hoc* analysis showed that P100 amplitude was larger for women than men (*t* = −2.368, *p* < 0.05, *Bonferroni corrected*). None of the other main effects {awareness [*F*_(2, 70)_ = 1.687, *p* = 0.193], emotion [*F*_(1, 35)_ = 1.71, *p* = 0.199], or hemisphere [*F*_(1, 35)_ = 2.567, *p* = 0.118]} was significant in P100 amplitude.

The two-way interaction between awareness and gender [*F*_(2, 35)_ = 3.602, *p* < 0.05] was significant statistically. None of the other gender-related interactions were significant {gender and emotion [*F*_(1, 35)_ = 3.316, *p* = 0.077], gender and hemisphere [*F*_(1, 35)_ = 0.156, *p* = 0.695]}. *Post-hoc* analyses showed that the difference between men and women was statistically significant in the subthreshold condition (*t* = −3.216, *p* < 0.05, *Bonferroni corrected*), but not in the near-threshold (*t* = −1.156, *p* = 0.125) or in the suprathreshold (*t* = −2.733, *p* = 0.068) conditions. Only in the subthreshold condition, women showed larger P100 amplitude compared to men.

**Figure 3:**
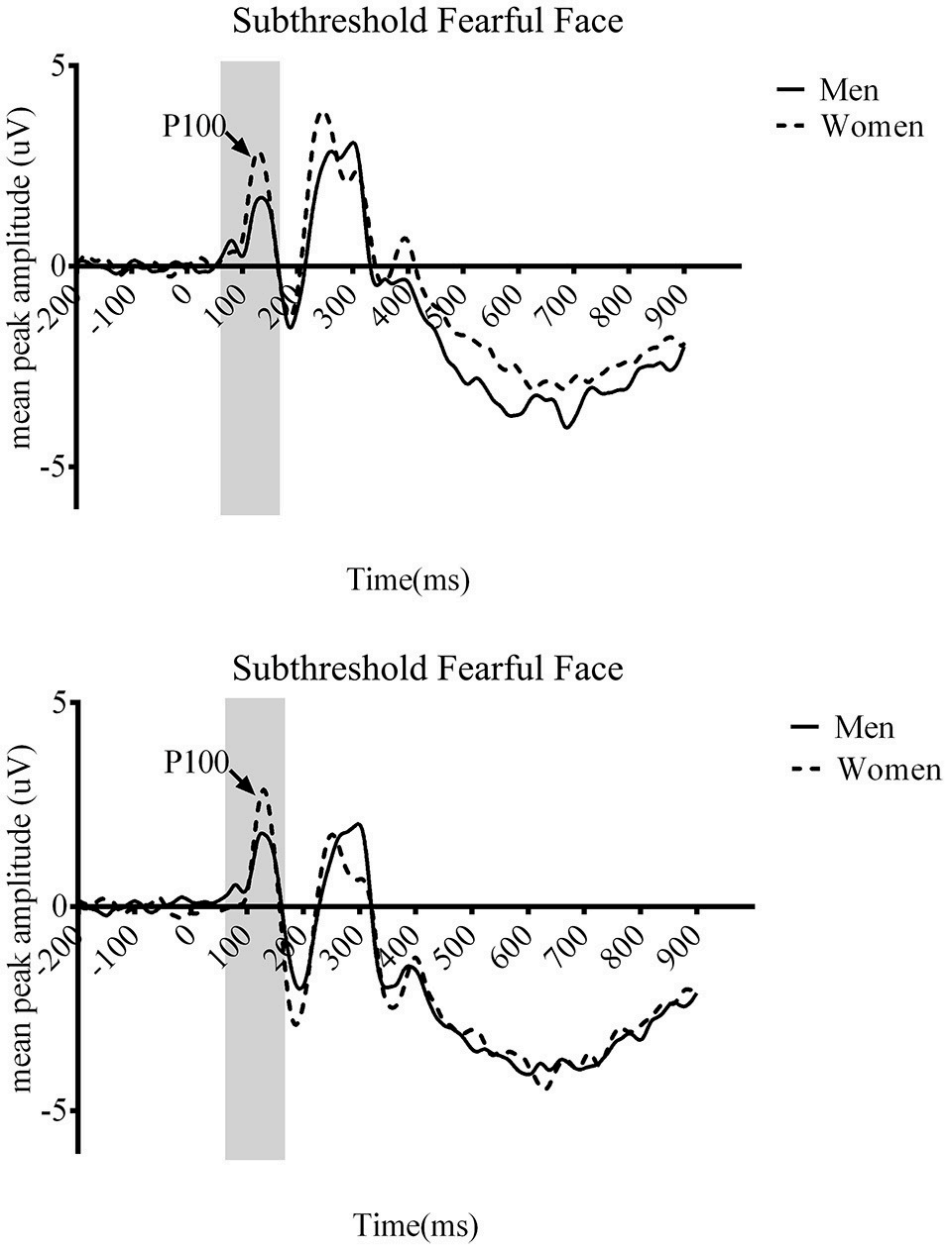
**Grand average event-related potential (ERP) waveforms of men (solid line) and women (dotted line) from the O1 (top) and O2 (bottom) in the main study**. The arrows indicate the P100 component.

We also found the significant three-way interaction across gender, awareness, and emotion [*F*_(1.422, 50.111)_ = 7.037, *p* < 0.01]. The other two gender-related three-way interactions were not significant {gender, awareness, and hemisphere [*F*_(1.432, 50.111)_ = 1.23, *p* = 0.29], gender, emotion, and hemisphere [*F*_(1, 35)_ = 0.01, *p* = 0.919]}. *Post-hoc* analyses revealed that significant gender difference for subthreshold fearful (*t* = −5.25, *p* < 0.01, see Figure 3) and near-threshold neutral face (*t* = −1.569, *p* < 0.05). No significant gender difference was observed for either fearful (*t* = −2.865, *p* = 0.062) or neutral (*t* = −2.601, *p* = 0.099) faces at suprathreshold condition. Women showed larger P100 amplitude particularly to fearful face processed at subthreshold compared to men. Also, P100 amplitude to neutral faces presented for near-threshold duration was also larger in women compared to men. The four-way interaction across gender, awareness, emotion, and hemisphere was not significant [*F*_(2, 70)_ = 0.178, *p* = 0.837].

In the P100 latency, none of the main effects nor interactions reached the level of statistical significance.

#### N170

Turning next to the N170 amplitude, there were no main effects of gender [*F*_(1, 35)_ = 1.677, *p* = 0.204], awareness [*F*_(1.134, 39.688)_ = 0.193, *p* = 0.289], emotion [*F*_(1, 35)_ = 0.563, *p* = 0.458], or hemisphere [*F*_(1, 35)_ = 0.263, *p* = 0.611]. There were no significant interactions between gender and awareness [*F*_(1.134, 39.688)_ = 1.256, *p* = 0.275], gender and emotion [*F*_(1, 35)_ = 0.146, *p* = 0.705], gender and hemisphere [*F*_(1, 35)_ = 0.174, *p* = 0.679], gender, awareness, and emotion [*F*_(2, 70)_ = 0.952, *p* = 0.391], gender, awareness, and hemisphere [*F*_(1.366, 47.827)_ = 2.086, *p* = 0.149], and gender, awareness, emotion, and hemisphere [*F*_(1.326, 46.405)_ = 0.376, *p* = 0.688]. However, there was significant interaction between emotion, hemisphere, and gender [*F*_(1, 35)_ = 4.427, *p* < 0.05]. Only women showed larger N170 amplitude at the left hemisphere to fearful faces compared to right hemisphere regardless of the awareness (*t* = 2.103, *p* < 0.05).

For N170 latency, there were no main effects of gender [F_(1, 35)_ = 0.003, *p* = 0.959], awareness [*F*_(1.396, 48.861)_ = 2.41, *p* = 0.117], emotion [*F*_(1, 35)_ = 0.081, *p* = 0.777], or hemisphere [*F*_(1, 35)_ = 0.419, *p* = 0.521]. Also, there were not significant interactions between gender and awareness [*F*_(1.396, 48.861)_ = 0.67, *p* = 0.873], gender and emotion [*F*_(1, 35)_ = 0.011, *p* = 0.916], gender and hemisphere [*F*_(1, 35)_ = 0.837, *p* = 0.366], gender, awareness, and emotion *F*_(2, 70)_ = 0.177, *p* = 0.838), and gender, awareness, emotion, and hemisphere[*F*_(2, 70)_ = 0.593, *p* = 0.555]. One of few significant three-way interactions was found between gender, awareness, and hemisphere [*F*_(1.374, 48.097)_ = 5.751, *p* < 0.05]. However, no significant gender difference was found in the *post-hoc* analysis. There was also significant interaction between gender, emotion, and hemisphere [*F*_(1, 35)_ = 7.29, *p* < 0.05].

#### EPN

None of the main or interaction effects were significant statistically either in the amplitude or in the latency of EPN.

#### N250

None of the main or interaction effects were significant statistically in the N250 amplitude.

In latency, there was no significant main effects of gender [*F*_(1, 35)_ = 1.127, *p* = 0.296], emotion [*F*_(1, 35)_ = 0.006, *p* = 0.99], or hemisphere [*F*_(2, 70)_ = 1.416, *p* = 0.25]. The only significant main effect was found for awareness [*F*_(1.579, 55.248)_ = 4.844, *p* < 0.05]. *Post-hoc* analysis showed that N250 latency in the near-threshold condition was longer than in the subthreshold condition (*t* = −7.536, *p* < 0.05), and N250 latency in the subthreshold condition was longer than in the suprathreshold condition (*t* = 16.854, *p* < 0.001). None of the interactions related to the gender factor was significant statistically.

#### P300

In the P300 amplitude, there was a significant main effect of emotion [*F*_(1, 35)_ = 5.471, *p* < 0.05]. However, there were no other significant main or interaction effects.

In the P300 latency, there was a significant main effect of emotion [*F*_(1, 35)_ = 9.483, *p* < 0.05] as well. However, none of the other main effects nor interaction effects reached the level of statistical significance.

### Depression control results

One might question whether the differences in the P100 amplitude between men and women in response to subthreshold fearful faces was derived mainly by the genuine gender difference, without intervention of anxiety or depressive symptoms. This question is not far-fetched based on previous studies (Mayer and Merckelbach, 1999; Kessler et al., 2005; Tan et al., 2011) and also based on the fact that some of the participants in the main experiment showed BAI and BDI scores above the healthy range. Therefore, we re-examined gender difference in ERP responses to subthreshold fearful faces by testing only those male and female participants whose BAI and BDI scores were within the healthy range.

From the main experiment, those participants whose BDI or BAI score were above 10 and 11, respectively, were excluded (BDI minimal range 0–9, BAI minimal range 0–10). As a result, two men and seven women were excluded. Ten participants (4 men, 6 women) were recruited additionally through online advertisement. Before participation, their BDI and BAI scores were pre-screened to ensure that they were not suffering from anxiety and depressive symptoms. With those additional participants, the age between gender (men: 28.90 ± 1.41 years old, women: 29.65 ± 1.75 years old) as well as education (men: 13.62 ± 0.38 years, women: 13.90 ± 0.369 years) remain indistinguishable (see Table 2).

**Table 2:** **Demographic characteristics and questionnaire scores of participants in the depression control experiment**.

	**Men (*n* = 21)**	**Women (*n* = 20)**	***t*-value**	***p* value**
Age (years)	28.9(±6.46)	29.65(±7.84)	−0.33	0.741
Education (years)	13.61(±1.74)	13.9(±1.65)	−0.52	0.599
STAI-trait	41.47(±5.47)	44.95(±4.85)	−2.14	0.038^*^
PANAS Score				
Positive	20.57(±4.83)	20.7(±5.96)	−0.07	0.939
Negative	21.85(±5.01)	22.65(±5.08)	−0.5	0.617
BAI	3 (±3.17)	5.25(±3.43)	−2.17	0.035^*^
BDI	4.95(±1.01)	6.5(±3.26)	−1.34	0.185

*Values are mean (±standard deviation) of each gender group*.

* *p < 0.05. STAI_trait, State and Trait Anxiety Inventory_trait; PANAS, Positive and Negative Affect Schedule; BAI, Beck's Anxiety Inventory; BDI, Beck's Depression Inventory*.

As a consequence of screening participants based on the scores of anxiety and depressive symptom questionnaires, BDI scores did not show any statistically significant difference between men and women [*t*_(39)_ = −1.34, *p* = 0.185], unlike in the main experiment (Table 2). BAI and STAI-trait scores, however, were still significantly different between men and women after excluding participants whose scores were outside the healthy ranges [BAI: *t*_(39)_ = −2.17, *p* < 0.05; STAI-trait: *t*_(39)_ = −2.14, *p* < 0.05]. We, therefore, took BAI and STAI-trait scores as covariants in the subsequent ERP data analyses. Scores of PANAS (either positive or negative) did not differ between men and women as in the main experiment.

**Figure 4:**
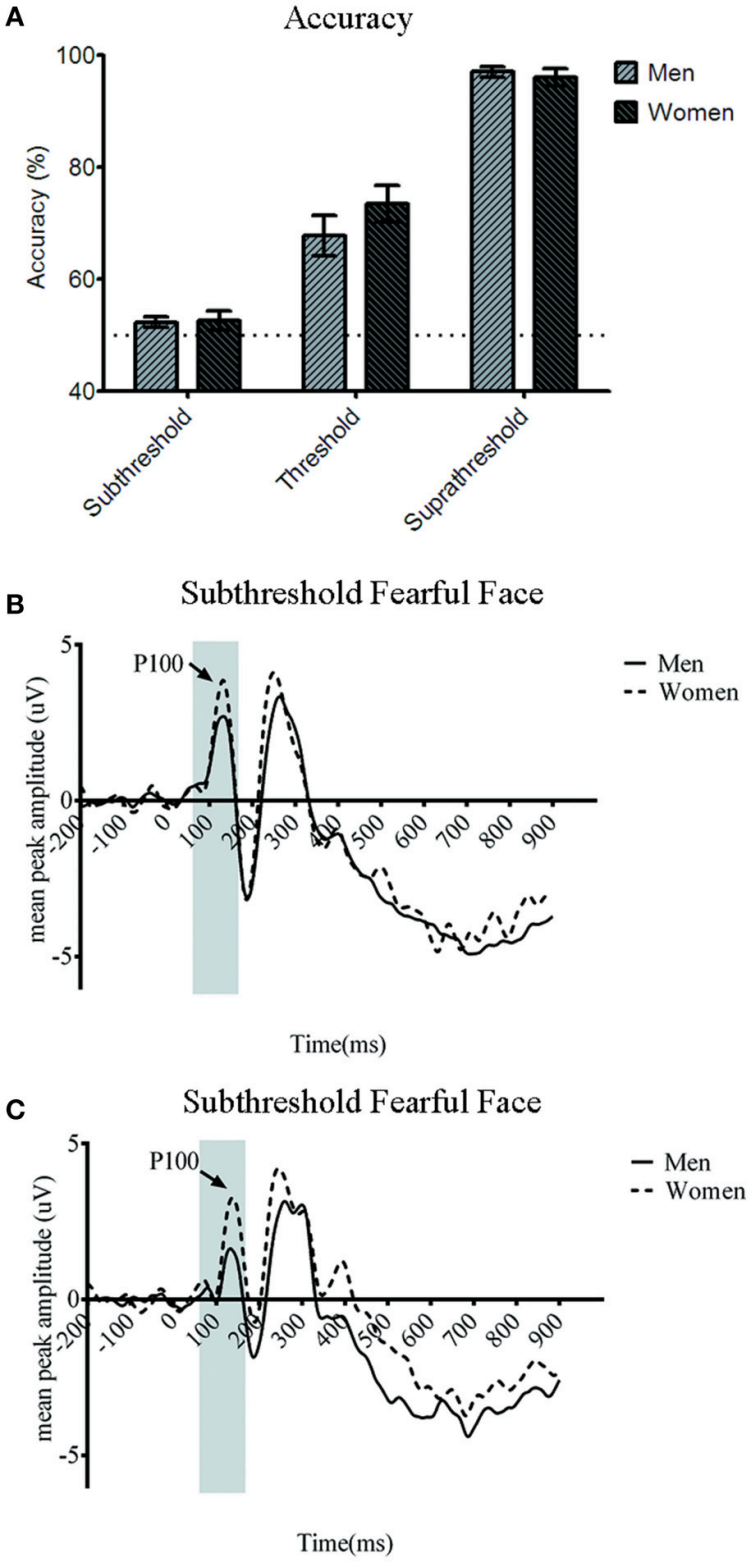
**(A)** Accuracy for three awareness conditions in the depression control. There was no significant gender difference of the accuracy for all three awareness conditions. Error bars denote ± standard error of mean. Note that the accuracy in the subthreshold condition was not different statistically from the chance level in both the gender groups. Grand average event-related potential (ERP) waveforms of men (solid line) and women (dotted line) from the **(B)** O1 and **(C)** O2 in the depression control.

The behavioral results echoed those in the main study. The emotion discrimination accuracy for the subthreshold condition was 52.15 ± 0.91% (SEM) for men and 53.63 ± 1.79% for women. For the near-threshold condition, men's accuracy was 69.9 ± 3.32% and women's was 71.8 ± 4.06%. In the suprathreshold condition, men's accuracy was 97.4 ± 0.79% and women's accuracy was 95.7 ± 1.62% (Figure 4A). The two-way mixed ANOVA analysis showed the main effect of awareness [*F*_(2, 78)_ = 210.336, *p* < 0.001] but no main effect of gender [*F*_(1, 39)_ = 0.058, *p* = 0.811] and no interaction between awareness and gender [*F*_(2, 78)_ = 0.042, *p* = 0.659]. Therefore, there was no gender-related difference in the emotion discrimination accuracy. One-sample *t*-test analysis showed that the accuracy in subthreshold condition did not differ significantly from the 50% chance level [men: *t*_(20)_ = 2.049, *p* = 0.054; women: *t*_(19)_ = 1.865, *p* = 0.078]. As in the main experiment, our manipulation of the awareness in the subthreshold condition was successful.

Turning finally to the P100 amplitude which is of most relevance to our purpose, the four-way ANOVA showed the significant main effect of gender [*F*_(1, 37)_ = 6.598, *p* < 0.05]. *Post-hoc* analysis showed that P100 amplitude was larger for women compared to men (*t* = −2.337, *p* < 0.05, *Bonferroni corrected*). None of the other main effects were significant statistically in P100 amplitude {awareness [*F*_(1.621, 60.01)_ = 1.678, *p* = 0.199], emotion [*F*_(1, 37)_ = 0.461, *p* = 0.501], or hemisphere [*F*_(1, 37)_ = 1.062, *p* = 0.31]}. We found no significant two-way interactions relevant to gender including the one between gender and awareness which was significant in the main study [*F*_(2, 74)_ = 1.499, *p* = 0.229], gender and emotion and gender [*F*_(1, 37)_ = 1.123, *p* = 0.296], and hemisphere and gender [*F*_(1, 37)_ = 0.359, *p* = 0.553]. However, the three-way interaction across gender, awareness, and emotion was statistically significant [*F*_(1.453, 53.772)_ = 4.536, *p* < 0.05], which replicated the results from the main study. *Post-hoc* analysis revealed gender difference for subthreshold fearful (*t* = −5.25, *p* < 0.001, *Bonferroni corrected*) and near-threshold neutral face (*t* = −1.569, *p* < 0.01). As in the main results from the participants some of whose anxiety scores were beyond the normal range, women showed larger P100 amplitude particularly to fearful faces processed subthreshold as well as to neutral faces in the near-threshold condition compared to men. No significant gender difference was observed for the subthreshold neutral (*t* = −1.54, *p* = 0.264), the near-threshold fearful (*t* = −1.21, *p* = 0.1), or suprathreshold faces wither fearful (*t* = −2.352, *p* = 0.074) and neutral (*t* = −2.55, *p* = 0.056). None of the other interaction effects was significant.

In the P100 latency, none of the main effects nor interactions reached the level of statistical significance.

## Discussion

In the main experiment, we have shown the gender difference of the early ERP responses to emotional face stimuli at the posterior electrode sites. Specifically, women showed larger amplitude of P100 component at O1 (Figure 4B) and O2 (Figure 4C) compared to men, which was only significant when they were presented with fearful faces too briefly so that it was processed at subthreshold. Such gender-related difference was not shown in the behavioral responses, in which both men and women showed a chance-level emotion discrimination performance. In an additional control experiment, with replacement of some participants whose BAI and BDI scores were beyond the healthy range, we replicated the gender difference in P100 amplitude in response to subthreshold fearful faces. Therefore, the larger P100 amplitude in women's posterior electrode sites than men's in response to emotional information processed at subthreshold was proven to be gender-specific, not mainly driven by clinically anxious or depressive personality factors.

The P100 ERP component is commonly thought to reflect low-level visual feature processing and be generated in early visual areas (Heinze et al., 1994). Therefore, the current results might be interpreted as gender differences in low-level visual processing in general. However, P100 has also been implicated in early face-specific processing (Itier and Taylor, 2004) even when low-level stimulus feature was controlled (Herrmann et al., 2005). Successful categorization of visual stimuli as faces was found to correlate with the early P100 component (Clark et al., 1996; Linkenkaer-Hansen et al., 1998; Itier and Taylor, 2002, 2004). The P100 component has also been implicated in emotion processing including correct detection of visual facial expressions (Utama et al., 2009). Amplitude of the P100 component is modulated by auditory emotional stimulus accompanied by visual emotional stimulus (Gerdes et al., 2014). Considering that the difference of the amplitude of P100 between men and women was observed only when the participants viewed fearful faces at subthreshold, our results are likely to reflect gender differences in visual processing of *emotional* information in faces.

We are not the first to report such gender-related P100 difference in emotional processing. Sass et al. (2010) have shown that a group of participants with self-reported high anxious arousal showed larger P100 than healthy controls in response to emotionally arousing words while the participants had to name the ink color of the word. Of more relevance to our current interest, women of high anxious arousal showed greater P100 than did men of high anxious arousal in response to emotional words including both threatening and pleasant ones. What makes the current results distinct from Sass et al. (2010) is the gender difference in P100 in response to emotional stimuli was observed when the stimuli were invisible.

In the current study, the gender difference in P100 responses was specific to the subthreshold fearful faces, not to the fearful faces viewed for longer durations and processed further. P100 has indeed been implicated in the subthreshold face processing in some previous studies. For example, Saito et al. (2007) have shown that the P100 amplitude in the occipital electrodes in response to faces was different significantly from the P100 amplitude in response to non-face stimuli when the stimuli were presented for 20 ms and processed outside conscious visual awareness. Moreover, the P100 amplitude was smaller for inverted faces than for upright faces when the face stimuli were processed at subthreshold duration. Such face-specialized P100 responses were not observed when the faces were presented for 30, or 300 ms, which were around and above the temporal threshold levels. These results suggest unconscious, face-specific processing reflected in the early P100 ERP component. Our results further extend the previous findings by showing P100's involvement in the unconscious *emotion-specific* face processing, which distinguishes women from men. Namely, the early automatic response to emotional information is amplified in women.

Individual differences in subthreshold emotional processing have also been addressed previously. A majority of those studies focused on neurological factor such as anxiety as the main source of individual differences, rather than gender, the focus of the current work. In one study, Li et al. (2007) tracked P100 responses of non-patient participants based on the degree of trait anxiety indicated by bispectral index (BIS) scores. Results showed a positive correlation between P100 amplitude associated with invisible emotional word and the trait anxiety. Specifically, the difference between P100 amplitude in response to threat-related words of subthreshold duration and P100 amplitude in response to neutral words of subthreshold duration was greater as the BIS score increased. Yet, in another study, the correlation was in the opposite direction; Walentowska and Wronka (2012) found a negative correlation between P100 amplitude and anxiety, implying that decreased early, and automatic sensitivity to subthreshold face in high anxious group. It is not our main concern to test these seemly contradictory results in previous studies. However, our results are suggestive of the apparent contradiction. There exist greater prevalence rates of anxiety disorders in women than in men (Cahill, 2006). Namely, our results showing the amplified P100 response to subthreshold fearful faces in women provide indirect evidence supporting the results of Li et al. (2007) reporting the positive relationship between P100 amplitude associated with invisible emotional word and the trait anxiety.

The tight coupling between gender and neuroticism factors is what requires great caution. A host of studies have shown the distinctive early stage of emotional processing exemplified by P100 in patients with schizophrenia (Javitt et al., 1993; Foxe et al., 2001; Campanella et al., 2012) and depression (Fotiou et al., 2003). This is of concern for most of the previous studies on gender differences in emotional processing, since other neuroticism factors might have covaried with gender. In a previous study, for example, N2 and P3 modulation only in women disappeared when depression, anxiety and alexithymia scores were matched between men and women in a modified emotional oddball task (Campanella et al., 2012). Further analysis has revealed that personality factors such as alexithymia were better at predicting the N2 latencies than the gender factor. It is also plausible that some personality factors lie at the core of what really distinguish men and women. Indeed, the main experiment of the current study showed statistically significant differences in STAI-trait and BAI, and BDI scores between men and women, despite the consideration of scores from the anxiety and depression questionnaires as covariates in the analyses. To disentangle these two sources of individual differences, most of the previous studies have attempted to show that patients with emotional disorders had automatic attentional bias toward negative emotions compared to healthy controls regardless of gender of the patients. For instance, patients with anxiety or depression had pre-attentive processing bias for anxiety- or depression-related words compared to healthy individuals regardless of the gender (Mogg et al., 1993). In contrast, the current study tried to tease the neuroticism factor from out of the gender factor by replacing the participants whose scores in those measures are higher than the healthy ranges with the new participants whose scores are within the healthy ranges in the depression control experiment. Since the greater P100 in women than in men in response to invisible emotional faces was replicated in the depression control experiment, we are more confident that the P100 difference between men and women is not entirely driven by the neuroticism factors. This last point is what makes the current work stand out amongst other previous works.

It should be noted, however, that the questionnaire scores, BAI and STAI-trait in particular, differed between men and women even when the acceptable range of anxiety and depression was controlled. Although the difference may have still affected the neurophysiological response even after it was controlled as a covariant, we believed that discarding the difference might incur rather an unnatural phenomenon. It is often accepted that men and women differ in emotional balance even in healthy population. The inherent gender difference in anxiety and depression level may be the natural phenomenon, therefore we did not try to artificially match scores for men and women. The current study demonstrated that there might be a biological difference between men and women in processing emotional information. However, relevant personality factors such as anxiety or depression could also be significant factors that cannot be separately considered. Future study should consider such personality factors when investigating gender difference in automatic response bias to emotional information.

In addition to P100, we found a few more significant results in terms of other ERP components.

First, there was hemisphere lateralization in N170 component regardless of awareness. N170 amplitude to fearful face was significantly larger at left hemisphere compared to right hemisphere only in women. This result is consistent with previous studies that observed dominance of left hemisphere to unpleasant stimuli in women and dominance of right hemisphere to pleasant stimuli in men. Several studies have observed gender difference of hemisphere lateralization at N170 component (Proverbio et al., 2006). A number of studies have shown right hemisphere dominance at N170 component to face stimuli especially when there are more men among the participants (Campanella et al., 2000; Itier and Taylor, 2004; Harris et al., 2005; Kovács et al., 2006). When there are more women among the participants, N170 activity seems to be either bilateral or more dominant at left hemisphere (Jemel et al., 2005; Meeren et al., 2005; Pourtois et al., 2005). In Righart and de Gelder study (2006), 10 among the 12 participants were women. They observed larger N170 amplitude to faces in fearful context compared to that in neutral context in the left hemisphere. Hemisphere lateralization is also observed in other components and brain areas. For instance, Gasbarri et al. (2007) observed enhanced P300 amplitude and latency to unpleasant pictures at left hemisphere for women while men showed enhanced P300 at right hemisphere. Although we did not find hemisphere lateralization for other components, it seems notable that only women showed left hemisphere dominance for face-specific component, which further indicates different brain mechanism of emotion processing between men and women.

Second, longer N250 latency to subthreshold compared to suprathreshold condition was observed in the current study. Several studies have suggested the correlation between task difficulty and longer N250 latency (Towey et al., 1980; Letourneau and Mitchell, 2008). To discriminate *invisible* facial expressions may have made decision-making more difficult for the participants resulting in longer N250 latency. However, it should be noted that we did not observe enhanced N250 amplitude or P300 amplitude to subthreshold fearful face compared to suprathrehsold fearful face. Previous studies have observed modulation of N250 and P300 complex by visibility of stimuli (Liddell et al., 2004; Williams et al., 2004; Kiss and Eimer, 2008). Although the awareness effect for these components was not our main interest, it may be taken into consideration that we did not find awareness effect for frequently observed components. The absence of differential activity N250 and P300 should be further investigated using different paradigms of making stimuli *invisible*.

The current study attempted to investigate the gender difference in processing emotional information at various levels of awareness. There exists a host of evidence that women are more responsive and expressive to emotional information compared to men, but the results from the current study demonstrated that healthy women have more intensive automatic response to negative emotional information at subthreshold level compared to men. This gender difference at early level of emotion processing (enhanced P100 amplitude to subthreshold fearful face in women) was observed even when depression and anxiety were controlled, implying further that women's vulnerability to the threats of depression and anxiety may be related to the different neurophysiological responses. Furthermore, faster and enhanced early response to subliminal emotional information in women is in line with how women tend to be more sensitive to stimulus in weaker intensity. Our results suggest that women's sensitivity to emotional stimuli which has been developed though evolution and social learning has biological bases. Even though there are some shortcomings in our study, such as the absence of gender difference at suprathreshold condition, this appears to be the first attempt to investigate gender difference of processing emotional information at subthreshold level of awareness in healthy subjects. To identify that this observation is not limited to a specific paradigm, a further study is needed such as using different paradigms inducing stimuli perceptually invisible.

## Author contributions

SAL suggested the idea, conducted the experiment, analyzed the results, and wrote the whole manuscript. MS helped the ERP analysis. SHL and CK designed the study, and edited the manuscript.

## Funding

This research was supported by the Brain Research Program through the National Research Foundation of Korea (NRF) funded by the Ministry of Science, ICT and Future Planning (NRF-2015M3C7A1028252). This research was also supported by the NRF funded by the Ministry of Education, Science and Technology (2013K2A1A2053850).

### Conflict of interest statement

The authors declare that the research was conducted in the absence of any commercial or financial relationships that could be construed as a potential conflict of interest.
